# Association between methyl donor nutrients and metabolic health status in overweight and obese adolescents

**DOI:** 10.1038/s41598-022-21602-9

**Published:** 2022-10-11

**Authors:** Donya Poursalehi, Keyhan Lotfi, Saeideh Mirzaei, Ali Asadi, Masoumeh Akhlaghi, Parvane Saneei

**Affiliations:** 1 Students’ Scientific Research Center, Isfahan University of Medical Sciences, Isfahan, Iran; 2 Department of Community Nutrition, School of Nutrition and Food Science, Nutrition and Food Security Research Center, Isfahan University of Medical Sciences, Isfahan, 81745-151 Iran; 3 Department of Community Nutrition, School of Nutritional Sciences and Dietetics, Tehran University of Medical Sciences, Tehran, Iran; 4 Department of Community Nutrition, School of Nutrition and Food Science, Shiraz University of Medical Sciences, Shiraz, Iran; 5 Department of Exercise Physiology, School of Physical Education and Sport Sciences, University of Tehran, Tehran, Iran

**Keywords:** Endocrine system and metabolic diseases, Paediatric research, Metabolic disorders, Epidemiology

## Abstract

Limited evidence is available regarding the association of methyl donor nutrients and adolescents’ metabolic health. Therefore, we investigated the relation between a combination of methyl donor nutrients and metabolic health status of overweight and obese Iranian adolescents. In this cross-sectional study, 203 overweight/obese adolescents were included, using a multistage cluster random-sampling method. Dietary intakes were assessed by a validated food frequency questionnaire. Methyl donor nutrient score (MDNS) was constructed based on deciles of vitamins B2, B6, B9, B12, methionine, choline and betaine. Glycemic profile, lipid profile, blood pressure and anthropometric indices were collected. Participants were classified as metabolically healthy obese or unhealthy obese (MUO) based on International Diabetes Federation (IDF) and IDF/Homeostasis Model Assessment Insulin Resistance (HOMA-IR) definitions. Mean age of adolescents was 13.98 $\pm$ 1.61 y and 50.2% of them was girls. After controlling all of the confounders, individuals in the top tertile of MDNS, had lower odds of MUO (OR 0.36; 95% CI 0.13–0.95) according to IFD criteria. Considering IDF/HOMA-IR criteria, an inverse marginally significant association was observed between the highest tertile of MDNS and odds of MUO (OR 0.36; 95% CI 0.12–1.02) in the fully-adjusted model. Furthermore, significant inverse association was found between each unit increase in MDNS and odds of MUO based on IDF criteria, but not for IDF/HOMA-IR definition. We found that overweight/obese adolescents with higher dietary intakes of methyl donor nutrients were less likely to be metabolically unhealthy. Further studies are needed to confirm the findings.

## Introduction

The rising prevalence of overweight and obesity among children and adolescents has been a challenging concern in the twenty-first century^1,2^. According to global estimates, there will be 268 and 145 million overweight and obese children and adolescents by 2025, respectively^3^. Furthermore, it has been estimated that about 4 million children will be overweight/ obese in Iran until 2025^4^. Obesity has been proven to physically and mentally affect children’s health, and it could lead to chronic diseases such as type 2 diabetes, hypertension and dyslipidemia^5^. Despite the considerable impact of adiposity, some obese individuals are known as metabolically healthy obese (MHO)^6^, while some others are considered as metabolically unhealthy obese (MUO)^6^. The developed criteria for categorizing obese individuals to MHO/MUO are mainly based on elevated blood pressure (BP), blood lipids and blood glucose, which are some components of metabolic syndrome (MetS)^7^.

Lifestyle factors such as diet along with genetic variables were notable determinants of developing childhood obesity and metabolic unhealthy profile^8^. Previous studies have shown inverse associations between healthy dietary patterns such as Mediterranean diet and dietary approach to stop hypertension (DASH) and metabolic healthy/unhealthy status^9,10^. In these healthy patterns, the emphasis is on high consumption of plant foods and low intake of red meats^9,10^, which might be independently related to metabolic disturbances^11–13^. Furthermore, it has been shown that MHO individuals have higher consumption of micronutrients such as calcium, potassium, magnesium and vitamins B group^10,14,15^.

Some nutrients including vitamins B2, B6, B9, B12, methionine, betaine and choline are defined as methyl donors due to their roles in a series of reactions known as the methylation cycle in which a methyl group can go on and attach to different molecules and regulate several processes^16^. Epigenetic mechanisms such as DNA methylation play an important role in leptin secretion as well as regulation of metabolic enzymes and can subsequently be involved in metabolic disorders^17^. Also, the conversion of homocysteine to methionine is dependent to the one-carbon methylation cycle, and disruption of this process can result in hyperhomocysteinemia and its related diseases^16^. Although no previous study has explored on methyl donor nutrients in relation to being MUO, some studies have explored the association between these nutrients and MetS and its components. Dietary folate intake has been observed to be inversely related to hypertension in women^18^, and MetS in both genders^19^. Also, a cross-sectional study in adults illustrated inverse associations between vitamins B6 and B9 intake and risk of MetS; but no significant association was observed with vitamin B12^20^. In contrast, in Mesoamerican adults, circulating vitamins B9 and B12 had positive associations with MetS, but vitamin B6 levels were inversely related to this outcome^21^. A study on Japanese children suggested that higher intakes of folate and vitamin B12 could be linked to reduced BP; however, no significant relation was observed for vitamin B6 intake^22^. Another cross-sectional investigation among Mesoamerican children has found a positive link between erythrocyte folate and MetS and a null association with B6 levels; whereas vitamin B12 levels were inversely associated with MetS^21^. Previous studies have investigated particular methyl donor nutrients in relation to metabolic status. As these nutrients are involved in a cyclic process, inadequacy of one of these nutrients might disrupt the whole methylation cycle. Therefore, it is worthwhile to consider the combination of these nutrients in relation to metabolic diseases. Furthermore, the mentioned studies have been mostly conducted in the Western countries with different dietary preferences from those in the Middle-East. Therefore, this study aimed to explore the association between dietary intakes of a combination of methyl donor nutrients and MUO profile in a sample of Iranian adolescents. In order to have enough MUO cases, we included only overweight and obese adolescents in the study. Our hypothesis was that an inverse association between dietary intake of methyl donor nutrients and being MUO might exist.

## Methods and materials

### Participants

A multi-level cluster random sampling method was used to choose adolescents (both girls and boys) aged from 12 to 18 years old for this cross-sectional study. The sample size of the current study was calculated based on previous published investigations^23,24^ that showed approximately 60% of overweight and obese adolescents suffer from MUO. Thus, with a power 80%, type I error of 0.05 (confidence interval of 0.95), and precision (d) of 7%, the minimum required sample size was calculated to be 188 subjects. Participants were randomly chosen from sixteen schools, which were located in five major regions of Isfahan, Iran. Body weight and height were assessed for all subjects, and body mass index (BMI) was determined by using the Quetelet formula (weight (kg)/ height^2^ (m)). Then, students were categorized as normal-weight, overweight, or obese based on the growth curve of age-sex-specific BMI percentiles^25^. Adolescents in overweight or obese category were considered for participation in this investigation. Individuals with various socioeconomic characteristics were included in our study. We did not include those who had a hereditary or endocrine disorder such as hypothyroidism, type I diabetes and Cushing's syndrome. Additionally, individuals on a special diet or those taking any medication that might alter their lipid profile, blood glucose, hypertension, and body weight were excluded. Also, those who were taking vitamin/mineral supplements were excluded from the study. Finally, 203 overweight/obese adolescents (102 girls and 101 boys) were included in the analysis. All participants signed informed consent forms. In addition, informed consents were obtained from their parents as few minors are involved in the study. The study protocol was also ethically approved by the Local Ethics Committee of the Isfahan University of Medical Sciences (IUMS).

### Assessment of dietary intakes

A validated 147-items food frequency questionnaire (FFQ) was applied to gather data on subjects' dietary intakes consumed in the last year^26,27^. This FFQ has been already validated among Iranian adolescents^26^. Also, documented associations between dietary intakes -obtained from this FFQ- and diseases or disorders in adolescents could be considered as an approach to validate this questionnaire^28^. Previous studies have shown that this FFQ could accurately show the relation between dietary intakes and several diseases among Iranian adolescents^26,29,30^. Consumption of each food item was questioned based on a daily, weekly, or monthly frequency and a standard portion size. Then, using household measurements, the reported portion size for each food item was converted to grams per day^31^. Nutritionist IV software was then used to compute nutrients’ intakes. This software has been developed based on USDA food composition database with some revisions for Iranian food.

### Methyl donor nutrients

Some earlier studies have suggested a score considering a combination of methyl donor nutrients^32–35^. Based on these previous studies^32–35^, vitamins B2, B6, B9, B12, betaine, choline, and methionine were considered as methyl donor nutrients. First, participants were divided into energy-adjusted deciles of each methyl donor nutrients intake. Participants in the first decile of each nutrient received a score of 1, whereas those in the last decile received a score of 10. Scores for the other deciles were distributed as well. To create a total methyl donor nutrient score (MDNS), we summed up each nutrient score for each subject. Final scores of MDNS for each participant ranged from 7 to 70.

### Assessment of anthropometric indices and cardio-metabolic risk factors

A trained researcher measured participants’ standing height to the nearest 0.1 cm without shoes by a stadiometer. Also, a calibrated electronic scale was used to measure weight in minimal clothing and without shoes (to the nearest 0.1 kg). After calculating BMI, participants were categorized as normal-weight (5th < BMI < 85th percentile), overweight (85th < BMI < 95th percentile), and obese (BMI > 95th percentile), based on the growth curve of age-sex-specific BMI percentiles^25^. To measure waist circumference (WC) to the nearest 0.1 cm, an un-stretched flexible anthropometric tape was used at a point midway between the lowest rib and the superior border of the iliac crest following a normal expiration and without putting any pressure to the surface of the body. Systolic blood pressure (SBP) and diastolic blood pressure (DBP) were taken by using a mercury sphygmomanometer with a suitable cuff size at the right arm after 15-min rest time. WC, SBP and DBP were measured twice for each person and finally the mean value of each item was considered in the analysis. Serum levels of triglycerides (TG), fasting blood glucose (FBG), high density lipoprotein cholesterol (HDL-c) and insulin were assessed using fasting blood samples. Homeostasis Model Assessment Insulin Resistance (HOMA-IR) was also calculated by following formula to determine Insulin resistance (IR)^36^: HOMA-IR = [(fasting insulin (mU/L) × FBG (mmol/L)]/22.5.

### Assessment of metabolic status

We used two different previously developed methods to categorize adolescents to MHO or MUO. In the first method -based on the modified International Diabetes Federation (IDF) criteria^37^- adolescents were identified as MUO subjects if they had two or more of the following abnormalities: (1) increased fasting blood glucose (≥ 100 mg/dL), (2) decreased HDL-c (< 40 mg/dL for the age of < 16 y, and < 50 mg/dL for girls/ < 40 mg/dL for boys in the ages of ≥ 16 y), (3) increased triglycerides (≥ 150 mg/dL) and (4) increased blood pressure (≥ 130/85 mmHg). Those with less than two of the above risk factors were considered as MHO subjects. In the second method, existence of IR was considered in addition to the IDF criteria. Therefore, participants having HOMA-IR scores ≥ 3.16 and ≥ 2 of IDF defined risk factors were classified as MUO cases, and those with HOMA-IR < 3.16 were known as MHO subjects^38^.

### Assessment of other variables

Physical activity level of the participants was gathered by using Physical Activity Questionnaire for Adolescents (PAQ-A). This questionnaire includes nine questions about various types of physical activity^39^. First eight items of PAQ-A were scored from 1 to 5; score of 1 indicated the lowest and score of 5 showed the highest level of physical activity in the last week. The last question assessed the unusual activity of participants in the preceding week. Finally, scores were summed up and adolescents were categorized as very active (score ≥ 4), active (score ≥ 3), low-active (3 < score ≤ 2), sedentary (or not having an orderly week activity) (score < 2). Only few participants were categorized as sedentary and very active; therefore, we combined sedentary with low-active level and active with very-active level to have two final categories of physical activity (low versus high). Socioeconomic status (SES) of the adolescents was examined by a validated demographic questionnaire^40^, including family size, parental job, parental education level, having cars in the family, having personal room, having computers/laptops and taking trips in the year. SES was then calculated to have a total score. A pre-tested questionnaire was also used to record the adolescents’ age, gender, medical history, medication and supplement use.

### Statistical analysis

In our study, dietary intake of each methyl donor nutrients was adjusted for total energy intake based on residual method and then MDNS was constructed. Adolescents were classified based on tertiles of energy-adjusted MDNS. For the general characteristics of subjects, continuous variables were reported as mean ± SD/SE, and categorical variables were reported as number and percentage. One-way analysis of variance (ANOVA) and chi-square test were respectively used for continuous and categorical variables to assess the differences of participants’ characteristics across the tertiles of MDNS. We also assess dietary intakes of participants across the tertiles of MDNS using analysis of covariance (ANCOVA). For total energy and macronutrients intakes, values were adjusted for age and sex, and the remaining nutrients and food groups were adjusted for age, sex and energy intake. For our primary analysis, the association between tertiles of MDNS and odds of being MUO was evaluated using binary logistic regression. Furthermore, we repeated the analysis considering the relation between each single methyl donor nutrient and MUO as well as combined methyl donor nutrients and each metabolic status components as our secondary analyses. The odds ratios (OR) and 95% confidence intervals (CI) for MUO status were computed in crude and adjusted models. In the first model, adjustments were done for some major confounders including age, gender and energy intake. In the second model, additional adjustments for socio-demographic variables such as physical activity level and SES were made. In the third model, dietary intakes of iron, niacin and saturated fatty acids intake were further adjusted based on previous literature^20,41,42^. In the last model, BMI was added to the adjustments, in order to have an independent association from obesity. In all models, the first tertile of MDNS was considered as the reference category. Tertile of MDNS was considered as an ordinal variable to assess the trend of odds ratios. Sensitivity analysis was also conducted by adjustment for alternative healthy eating index (AHEI-2010), as a healthy diet quality index, instead of above mentioned nutrients in the third model. We have additionally evaluated the chance of being MUO for each unit increase in MDNS. We used SPSS software version 26 (IBM, Chicago, IL) for all the analysis. P-values < 0.05 were considered as statistically significant.

### Ethical approval and consent to participate

The study procedure was performed according to declaration of Helsinki and STROBE checklist. All participants provided informed written consent. The study protocol was approved by the local Ethics Committee of Isfahan University of Medical Sciences. Informed consent was obtained from all participants involved in the study.

## Results

The study sample consisted of 203 overweight or obese adolescents with a mean age of 13.98 $\pm$ 1.61 years and a mean weight of 73.48 $\pm$ 11.60 kg. About 50.25% of the study population was girls. General characteristics of study participants across tertiles of MDNS are provided in Table 1. Adolescents in the last tertile of MDNS, compared with the first tertile, were more likely to be physically active and had higher HDL cholesterol levels. Furthermore, they had lower FBG, insulin, HOMA-IR index, and triglyceride concentrations. However, there were no significant differences in age, weight, BMI, gender, SES, SBP and DBP among tertiles of MDNS.

**Table 1:** General characteristics of study participants across tertiles of the mehtyl donor nutrients score (n = 203)^1^.

	Tertiles of energy-adjusted MDNS^2^			
	T_1_ (n = 67)	T_2_ (n = 70)	T_3_ (n = 66)	P^3^
Crude MDNS range	< 12	12–66	> 66	
Energy-adjusted MDNS range	< 32	32–47	> 47	
MDNS	22.49 $\pm$ 5.16	39.36 $\pm$ 4.63	53.89 $\pm$ 4.10	< 0.001
Age (y)	13.99 $\pm$ 1.48	13.86 $\pm$ 1.52	14.11 $\pm$ 1.82	0.67
Weight (kg)	76.08 $\pm$ 11.99	71.45 $\pm$ 11.26	73.00 $\pm$ 11.23	0.06
Body mass index (kg/m^2^)	27.89 $\pm$ 3.09	27.05 $\pm$ 2.98	27.14 $\pm$ 3.61	0.26
**Overweight/obesity prevalence, n (%)**				0.60
Overweight	31 (46.3)	37 (52.9)	36 (54.5)	
Obese	36 (53.7)	33 (47.1)	30 (45.5)	
Waist circumference (cm)	91.82 $\pm$ 7.81	89.66 $\pm$ 7.27	89.53 $\pm$ 8.61	0.17
**Gender, n (%)**				0.35
Boy	35 (52.2)	30 (42.9)	36 (54.5)	
Girl	32 (47.8)	40 (57.1)	30 (45.5)	
**Physical activity levels, n (%)**				< 0.001
Low^4^	48 (71.6)	37 (52.9)	17 (25.8)	
High^5^	19 (28.4)	33 (47.1)	49 (74.2)	
**Socioeconomic status levels, n (%)**				0.28
Low	23 (34.3)	23 (32.9)	13 (19.7)	
Moderate	30 (44.8)	28 (40.0)	32 (48.5)	
High	14 (20.9)	19 (27.1)	21 (31.8)	
Systolic blood pressure (mmHg)	115.70 $\pm$ 17.25	112.20 $\pm$ 17.42	110.20 $\pm$ 20.16	0.22
Diastolic blood pressure (mmHg)	74.68 $\pm$ 13.13	72.80 $\pm$ 10.51	73.03 $\pm$ 10.37	0.58
Fasting blood glucose (mg/dL)	100.70 $\pm$ 8.65	98.36 $\pm$ 8.72	95.29 $\pm$ 7.30	0.01
Insulin (µUI/mL)	24.39 $\pm$ 15.27	20.10 $\pm$ 9.26	16.74 $\pm$ 11.83	0.02
HOMA-IR index	6.06 $\pm$ 3.76	4.93 $\pm$ 2.46	4.06 $\pm$ 3.26	0.02
Triglycerides (mg/dL)	143.24 $\pm$ 73.84	119.47 $\pm$ 55.21	102.97 $\pm$ 64.46	0.02
HDL cholesterol (mg/dL)	42.81 $\pm$ 7.94	44.89 $\pm$ 7.82	46.80 $\pm$ 7.62	0.01

^1^All values are means ± standard deviation (SD), unless indicated.

^2^MDNS components were adjusted for energy intake based on residual method.

^3^Obtained from ANOVA for continuous variables and chi-square test for categorical variables.

^4^Includes sedentary and low-active participants.

^5^Includes active and very-active participants.

Table 2 indicates dietary intakes of selected food groups and nutrients of subjects across tertiles of MDNS. Participants in the third tertile of MDNS in comparison to the first category were more likely to have higher consumption of fruits, vegetables, meats, fish, legumes, nuts, dairy and lower consumption of grains. Also, individuals in the top tertile of MDNS, compared with the bottom, had higher intakes of proteins, fats, dietary fiber and saturated fats and lower intakes of carbohydrates, vitamin B1, vitamin B3 and iron. No substantial differences for omega-3 fatty acids intake was observed between tertiles of MDNS.

**Table 2:** Multivariable-adjusted intakes of selected food groups and nutrients of study participants across tertiles of methyl donor nutrients score (n = 203)^1^.

	Tertiles of energy-adjusted MDNS^2^			P^3^
	T_1_ (n = 67)	T_2_ (n = 70)	T_3_ (n = 66)	
Energy (Kcal/d)	2946.61 $\pm$ 66.32	2838.61 $\pm$ 65.30	2865.59 $\pm$ 67.07	0.49
**Food groups (g/day)**				
Fruits	242.65 $\pm$ 18.41	355.10 $\pm$ 18.10	399.73 $\pm$ 18.58	< 0.001
Vegetables	174.47 $\pm$ 18.06	259.69 $\pm$ 17.77	396.53 $\pm$ 18.23	< 0.001
Meats	59.05 $\pm$ 3.94	72.57 $\pm$ 3.87	74.34 $\pm$ 3.97	0.01
Fish	4.44 $\pm$ 0.81	7.39 $\pm$ 0.79	11.44 $\pm$ 0.81	< 0.001
Legumes	34.44 $\pm$ 3.30	48.38 $\pm$ 3.25	64.16 $\pm$ 3.33	< 0.001
Nuts	7.09 $\pm$ 1.27	13.29 $\pm$ 1.25	16.16 $\pm$ 1.28	< 0.001
Grains	764.56 $\pm$ 14.84	639.53 $\pm$ 14.59	563.17 $\pm$ 14.97	< 0.001
Dairy	341.11 $\pm$ 20.72	524.89 $\pm$ 20.38	682.63 $\pm$ 20.91	< 0.001
**Other nutrients**				
Proteins (% of energy)	12.65 $\pm$ 0.17	14.12 $\pm$ 0.17	16.20 $\pm$ 0.17	< 0.001
Fats (% of energy)	27.56 $\pm$ 0.63	29.73 $\pm$ 0.62	29.22 $\pm$ 0.64	0.04
Carbohydrates (% of energy)	60.89 $\pm$ 0.59	57.63 $\pm$ 0.59	56.35 $\pm$ 0.60	< 0.001
Dietary fiber (g/d)	16.29 $\pm$ 0.50	19.33 $\pm$ 0.49	22.77 $\pm$ 0.51	< 0.001
Omega-3 fatty acids (g/d)	0.60 $\pm$ 0.02	0.60 $\pm$ 0.02	0.61 $\pm$ 0.02	0.99
Vitamin B1 (mg/d)	2.77 $\pm$ 0.04	2.60 $\pm$ 0.04	2.57 $\pm$ 0.04	< 0.001
Vitamin B3 (mg/d)	28.93 $\pm$ 0.41	26.96 $\pm$ 0.40	26.84 $\pm$ 0.41	< 0.001
Iron (mg/d)	26.85 $\pm$ 0.64	24.08 $\pm$ 0.63	23.90 $\pm$ 0.65	0.01
Saturated fat (mg/d)	24.58 $\pm$ 0.67	27.94 $\pm$ 0.66	29.55 $\pm$ 0.67	< 0.001

^1^All values are means ± standard error (SE); energy and macronutrients intake is adjusted for age and gender; all other values are adjusted for age, gender and energy intake.

^2^MDNS components were adjusted for energy intake based on residual method.

^3^Obtained from ANCOVA.

**Figure 1:**
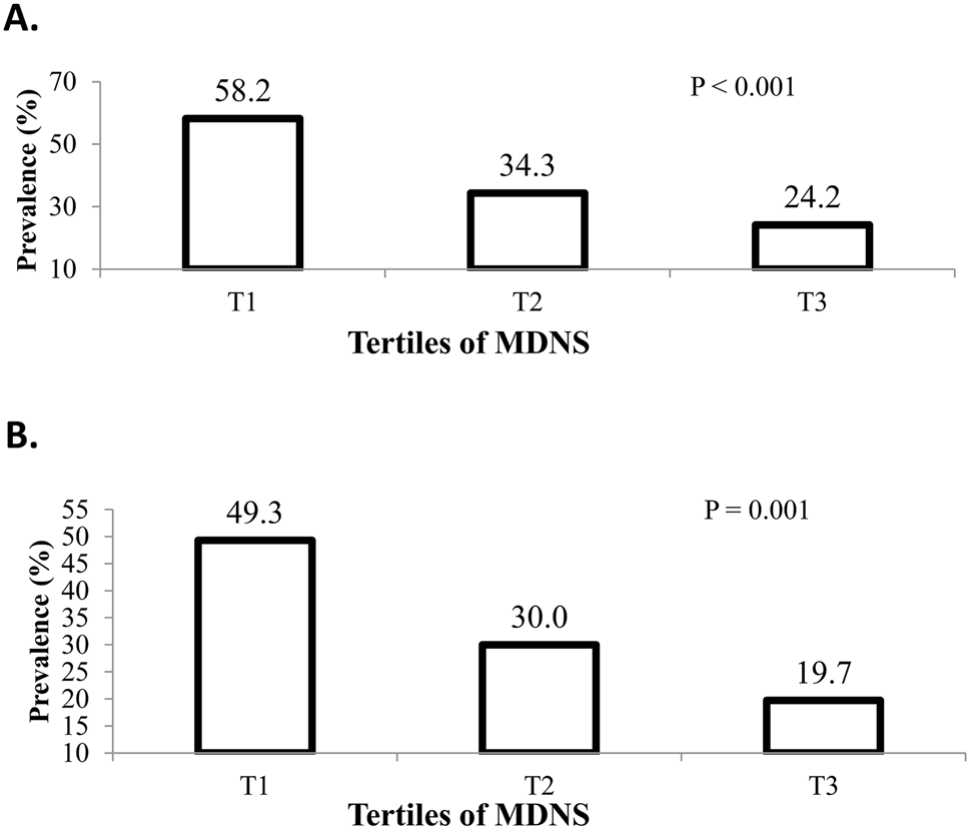
Prevalence of MUO across tertiles of MDNS in the study population. (**A**) MUO based on IDF definition. (**B**) MUO based on IDF/HOMA-IR definition.

As shown in Fig. 1, based on the IDF criteria, adolescents at the top tertile of MDNS, in comparison with the reference group, had a lower prevalence of being MUO (24.2% vs. 58.2%; P $<$ 0.001). Similarly, by considering the IDF/HOMA-IR criteria, the prevalence of being MUO in the last tertile of MDNS was lower than the first tertile (19.7% vs. 49.3%; P = 0.001).

Crude and multivariable-adjusted odds ratios for being MUO across tertiles of MDNS are reported in Table 3. According to the IDF definition, adolescents in the third tertile of MDNS compared to the first category, had a 77% decreased odds of being MUO in the crude model (OR 0.23; 95% CI 0.11–0.48). After adjusting for all potential confounders, such association was attenuated but still significant (OR 0.36; 95% CI 0.13–0.95). When the IDF/HOMA-IR criteria was considered for defining MUO, a significant inverse association was observed between MDNS and odds of being MUO in the crude model (OR for T3 vs. T1: 0.25; 95% CI 0.12–0.55) and after controlling for age, sex and energy intake (OR for T3 vs. T1: 0.27; 95% CI 0.12–0.61). However, after taking all potential confounders into account, the association became marginally significant (OR for T3 vs. T1: 0.36; 95% CI 0.12–1.02). Considering MDNS as a continuous variable, a significant inverse association was seen between each unit increase in MDNS and odds of MUO based on IDF criteria in the maximally-adjusted model (OR 0.96; 95% CI 0.93–0.99). However, this association was not significant based on IDF/HOMA-IR criteria (OR 0.96; 95% CI 0.93–1.10).

**Table 3:** Multivariable-adjusted odds ratio for MUO across tertiles of methyl donor nutrients score (n = 203)^1^.

	Tertiles of energy-adjusted MDNS^2^				Per 1 unit increase
	T_1_ (n = 67)	T_2_ (n = 70)	T_3_ (n = 66)	P_trend_	
**MUO Based on IDF criteria**					
MUO cases (n)	39	24	16		
Crude	1.00	0.38 (0.19–0.75)	0.23 (0.11–0.48)	< 0.001	0.95 (0.93–0.98)
Model 1	1.00	0.42 (0.20–0.86)	0.24 (0.11–0.53)	< 0.001	0.96 (0.93–0.98)
Model 2	1.00	0.54 (0.24–1.19)	0.50 (0.20–1.21)	0.10	0.98 (0.95–1.00)
Model 3	1.00	0.42 (0.18–0.98)	0.36 (0.13–0.95)	0.03	0.96 (0.93–0.99)
Model 4	1.00	0.42 (0.18–0.99)	0.36 (0.13–0.95)	0.03	0.96 (0.93–0.99)
**MUO Based on IDF/HOMA-IR criteria**					
MUO cases (n)	33	21	13		
Crude	1.00	0.44 (0.22–0.89)	0.25 (0.12–0.55)	< 0.001	0.96 (0.94–0.98)
Model 1	1.00	0.53 (0.25–1.10)	0.27 (0.12–0.61)	0.002	0.96 (0.94–0.98)
Model 2	1.00	0.72 (0.32–1.62)	0.54 (0.21–1.39)	0.19	0.98 (0.95–1.01)
Model 3	1.00	0.56 (0.24–1.33)	0.37 (0.13–1.03)	0.05	0.96 (0.93–1.10)
Model 4	1.00	0.57 (0.24–1.35)	0.36 (0.12–1.02)	0.05	0.96 (0.93–1.10)

^1^All values are odds ratios and 95% confidence intervals. Model 1: Adjusted for age, gender, energy intake. Model 2: More adjustments for physical activity levels, socioeconomic status. Model 3: Further adjustments for iron, niacin, saturated fatty acids. Model 4: More adjustment for BMI.

^2^MDNS components were adjusted for energy intake based on residual method.

As shown in Supplemental Table 1, sensitivity analysis indicated that after further adjustment for the overall diet quality index (AHEI-2010), instead of dietary nutrients in the third model, no significant association was observed between MDNS and odds of MUO based on IDF or IDF/HOMA-IR criteria. Further adjustment for BMI in the last model did not change the findings.

Crude and multivariable-adjusted odds ratios for metabolic disturbances across tertiles of MDNS are reported in Supplemental Table 2. Compared to the first tertile of MDNS, individuals in the third tertile were less likely to have high FBS, TG and HOMA-IR, after adjustments for all potential confounders. However, no significant association was observed between MDNS and low HDL-c or high BP.

Crude and multivariable-adjusted odds ratios for MUO across tertiles of each individual methyl donor nutrients are provided in Supplemental Table 3 and 4. Significant inverse associations were found between the highest intake of B2, B12, choline and methionine and MUO based on IDF definition (Supplemental Table 4). Considering IDF/HOMA-IR criteria, significant associations were seen between B12, choline and methionine and MUO (Supplemental Table 4). The relations of other nutrients and MUO were not significant.

## Discussion

Our results suggested that higher consumptions of methyl donor nutrients had an inverse association with being MUO based on IDF criteria. This relationship was significant even after controlling all of the covariates. Considering IDF/HOMA-IR criteria, we also found an inverse association between dietary intakes of methyl donor nutrients with being MUO. However, after considering the potential confounders, the association was attenuated. Sensitivity analysis revealed no significant association after considering the overall diet quality as a potential cofounder. This may be due to the fact that probably individuals with more consumption of methyl donor nutrients had a healthy and high quality dietary pattern. In addition, MDNS was inversely related to elevated FBS, TG and HOMA-IR. Furthermore, intake of B2, B12, choline and methionine was inversely related to MUO, based on IDF criteria. Similarly, B12, choline and methionine intake was negatively associated with MUO, based on IDF/HOMA-IR definition. The novelty of our study was exploring the association between combined methyl donor nutrients and metabolic health status as well as their single components among overweight or obese adolescents for the first time.

Individuals with MUO are at a higher risk of chronic conditions such as cardiovascular diseases^43^. Therefore, to reduce the obesity crisis, preventing and reversing the transition from MHO to MUO would be an important goal^2^. We observed that dietary intake of methyl donor nutrients was inversely associated with being MUO. Thus, consuming these nutrients or rich food sources of them might be an effective clinical advice for adolescents to prevent obesity-related metabolic comorbidities.

According to our findings, a higher intake of methyl donor nutrients was associated with lower odds of being MUO in adolescents. Moreover, high consumption of methyl donor nutrients was inversely associated with elevated FBS, TG and HOMA-IR. The magnitude of risk reduction was higher for HOMA-IR and FBS. Also, among methyl donor nutrients, vitamin B12 had the strongest association with MUO risk reduction. Limited evidence is available regarding methyl donor nutrients and metabolic disorders among children and adolescents. A cross-sectional study conducted among Japanese preschool children (n = 418, aged 3–6 years) found that a higher consumption of vitamin B12 was associated with 6.5 and 5.7 mmHg lower SBP and DBP, respectively^22^. This study also indicated 4.1 mmHg lower SBP in relation to higher dietary intakes of folate, but no significant link was revealed between vitamin B6 intake and BP^22^. Another report from THUSA BANA study, that was conducted among 321 black boys and 371 girls with 10–15 years of age, found that dietary folate intake could contribute to the etiology of hypertension^44^.

The discrepancies between our findings and the mentioned studies could be due to the differences in study design, studied populations, measurement tools, and also different confounding variables in the analysis. Our study was performed among overweight and obese adolescents, while most previous studies were conducted among subjects with different BMI categories. Furthermore, nutrients fortification varied widely between different countries that could probably affect the results. In addition, these studies have considered a single nutrient as the exposure and a single metabolic risk factor as the outcome of interest. However, we explored the combination of methyl donor nutrients in relation to the existence of ≥ 2 metabolic disturbances. It must be kept in mind that nutrients together could have some synergistic effects on the outcome of interest. Due to the lack of studies on the relation between methyl donor nutrients intake and metabolic abnormalities, further well-designed prospective studies are warranted.

In addition to dietary intakes of methyl donor nutrients, circulating levels of these nutrients were also evaluated in some investigations. A cross-sectional study among 237 school-age children (7–12 years) from nine Mesoamerican countries revealed that metabolic risk score was inversely related to plasma vitamin B12 levels, and positively associated with erythrocyte folate^21^. However, no substantial association was observed in case of vitamin B6 levels^21^. Another cross-sectional study among 256 Turkish children revealed that vitamin B12 concentrations were lower in obese children with MetS than in those without MetS^45^. On the other hand, Dursun et al. in their cross-sectional study found an inverse association between vitamin B12 levels and insulin resistance in obese adolescents^46^. Similarly, Ho et al. have documented a significant number of adolescents who were at risk of type 2 diabetes had vitamin B12 deficiency^47^.

Mechanisms by which methyl donor nutrients might be protective against MUO could be explained by their role in each component of metabolic health status. It has been shown that dietary intakes of vitamins B6, B9 and B12 could decrease plasma homocysteine levels^48^. These nutrients are key factors in the conversion of homocysteine to cysteine^48^. Therefore, inadequate methyl donor nutrients consumption may lead to elevated homocysteine concentrations^49^. It has been suggested that elevated homocysteine can increase oxidative stress and endothelial dysfunction that can lead to vascular stiffness and finally result in high BP^50^. Also, elevated homocysteine and its subsequent inflammation may have deleterious effects on beta-cells function and insulin resistance leading to increased blood glucose^16^. Another possible mechanism is related to the role of methylation reactions in the synthesis of serotonin. Serotonin can stimulate endothelial nitric oxide synthase and have a positive impact on endothelial function^51^. Folate may also have direct effect on nitric oxide production and regulation of blood pressure^52^. In addition, it has been documented that vitamin B2 deficiency can promote the release of inflammatory cytokines in adipose tissue accompanied by hyperleptinemia and hypoadiponectinemia^53^. These disturbances are all associated with an increased risk of insulin resistance and chronic inflammation as well as obesity^53^. Methyl donor nutrients can also regulate insulin secretion and glycemic control by reducing oxidative stress and systemic inflammation^54^. The process of methylation in gene expression is additionally responsible for leptin secretion involved in regulating satiety and obesity as well as metabolic diseases^17^.

Our study has several strengths. It was the first study in which a sample of Iranian adolescents was investigated for the relation between a combination of methyl donor nutrients and metabolic status. Moreover, all measurements were performed by trained nutritionists that increased the accuracy of the assessment. Numerous potential confounders were also considered in the analysis. Nevertheless, our study has also some limitations that deserve to mention. The cross-sectional design of the study did not allow us to determine causal relations. Therefore, prospective cohort studies are required to verify the causal relationship between methyl donor nutrients intake and metabolic healthy status. Despite the use of a validated FFQ for assessment of dietary intakes, possible misreporting of participants is unavoidable. In addition, although the MDNS has not been not formally validated in our study population, some previous studies have documented the relation between this score and various diseases, such as breast cancer^34,35^, and psychological disorders (depression, anxiety, distress)^32^; so, these evidences can be considered as an equivalent strategy to validate this score^28^. Furthermore, even after controlling for a variety of confounders, some residual variables could affect the results. Despite some studies on the relationship between methyl donor nutrients and non-communicable diseases, there is no available biomarker for assessing the overall methyl donor nutrient intake or body status; more studies are needed to find such an applicable biomarker. Current study was performed among adolescents in a developing country; therefore, generalization of our findings to other nations should be done with caution.

In conclusion, we found that overweight and obese adolescents with higher consumption of methyl donor nutrients were less likely to be MUO considering both IDF and IDF/HOMA-IR definitions. Therefore, our study has clinical importance for public health. Further prospective studies are needed confirm our findings. Also, clinical trials are warranted to assess the potential effect of methyl donor nutrients on cardio metabolic risk factors among adolescents.

## Supplementary Information

Supplementary Information.

## Data Availability

The data that support the findings of this study are available from the corresponding author upon reasonable request.

## Abbreviations

AHEI: Alternative healthy eating index
ANCOVA: Analysis of covariance
ANOVA: Analysis of variance
BP: Blood pressure
BMI: Body mass index
95% CI: 95% Confidence interval
DASH: Dietary approaches to stop hypertension
DBP: Diastolic blood pressure
TG: Triglycerides
FBG: Fasting blood glucose
FFQ: Food frequency questionnaire
HDL-c: High density lipoprotein cholesterol
HOMA-IR: Homeostasis model assessment insulin resistance
IDF: International Diabetes Federation
IR: Insulin resistance
MetS: Metabolic syndrome
MDNS: Methyl donor nutrient score
MHO: Metabolically healthy obese
MUO: Metabolically unhealthy obese
OR: Odds ratios
PAQ-A: Physical activity questionnaire for adolescents
SBP: Systolic blood pressure
SD: Standard deviation
SE: Standard error
SES: Socioeconomic status
TG: Triglycerides
SPSS: Statistical package for the social sciences
WC: Waist circumference
WHO: World Health Organization
