# Supplementary material for: Association between methyl donor nutrients and metabolic health status in overweight and obese adolescents
Source: Sci Rep. 2022 Oct 11;12:17045. doi: 10.1038/s41598-022-21602-9 (PMC9554193; doi:10.1038/s41598-022-21602-9)
Supplement: Supplementary file 1 — Supplementary Information. [file 41598_2022_21602_MOESM1_ESM.docx]

**Supplemental Table 1. Multivariable-adjusted odds ratio for MUO across tertiles of methyl donor nutrients score (n=203)^1^**

|  | Tertiles of energy-adjusted MDNS^2^ | | | | Per 1 unit increase |
| --- | --- | --- | --- | --- | --- |
|  | T_1_  (n=67) | T_2_  (n=70) | T_3_  (n=66) | P_trend_ |  |
| ***MUO Based on IDF criteria*** |  |  |  |  |  |
| MUO cases (n) | 39 | 24 | 16 |  |  |
| Crude | 1.00 | 0.38 (0.19-0.75) | 0.23 (0.11-0.48) | <0.001 | 0.95 (0.93-0.98) |
| Model 1 | 1.00 | 0.42 (0.20-0.86) | 0.24 (0.11-0.53) | <0.001 | 0.96 (0.93-0.98) |
| Model 2 | 1.00 | 0.54 (0.24-1.19) | 0.50 (0.20-1.21) | 0.10 | 0.98 (0.95-1.00) |
| Model 3 | 1.00 | 0.58 (0.25-1.35) | 0.63 (0.24-1.67) | 0.29 | 0.98 (0.95-1.01) |
| Model 4 | 1.00 | 0.59 (0.25-1.38) | 0.62 (0.24-1.65) | 0.28 | 0.98 (0.95-1.01) |
| ***MUO Based on IDF/HOMA-IR criteria*** |  |  |  |  |  |
| MUO cases (n) | 33 | 21 | 13 |  |  |
| Crude | 1.00 | 0.44 (0.22-0.89) | 0.25 (0.12-0.55) | <0.001 | 0.96 (0.94-0.98) |
| Model 1 | 1.00 | 0.53 (0.25-1.10) | 0.27 (0.12-0.61) | 0.002 | 0.96 (0.94-0.98) |
| Model 2 | 1.00 | 0.72 (0.32-1.62) | 0.54 (0.21-1.39) | 0.19 | 0.98 (0.95-1.01) |
| Model 3 | 1.00 | 0.81 (0.35-1.90) | 0.68 (0.24-1.88) | 0.44 | 0.99 (0.96-1.02) |
| Model 4 | 1.00 | 0.83 (0.35-1.95) | 0.64 (0.23-1.79) | 0.39 | 0.99 (0.96-1.02) |

^1^All values are odds ratios and 95% confidence intervals. Model 1: Adjusted for age, gender, energy intake. Model 2: More adjustments for physical activity levels, socioeconomic status. Model 3: Further adjustments for diet quality index (AHEI.2010). Model 4: More adjustment for BMI.

^2^MDNS components were adjusted for energy intake based on residual method.

**Supplemental Table 2. Multivariable-adjusted odds ratio for metabolic components across tertiles of MDNS (n=203) ^1^**

|  | Tertiles of energy-adjusted MDNS^3^ | | |  |
| --- | --- | --- | --- | --- |
|  | T_1_  (n=67) | T_2_  (n=70) | T_3_  (n=66) | P_trend_ |
| **Hyperglycemia (FBS ≥ 100 mg/dL)** |  |  |  |  |
| Crude | 1.00 | 0.35 (0.18-0.70) | 0.19 (0.09-0.40) | <0.001 |
| Multivariable-adjusted^2^ | 1.00 | 0.31 (0.14-0.70) | 0.16 (0.06-0.42) | <0.001 |
| **Hypertriglyceridemia (TG ≥150mg/dL)** |  |  |  |  |
| Crude | 1.00 | 0.63 (0.30-1.30) | 0.23 (0.10-0.56) | 0.01 |
| Multivariable-adjusted^2^ | 1.00 | 0.84 (0.36-1.93) | 0.30 (0.10-0.89) | 0.04 |
| **Low-HDL cholesterolemia^4^** |  |  |  |  |
| Crude | 1.00 | 0.48 (0.24-0.95) | 0.39 (0.19-0.80) | 0.01 |
| Multivariable-adjusted^2^ | 1.00 | 0.55 (0.23-1.34) | 1.05 (0.39-2.82) | 0.96 |
| **Hypertension (BP ≥130/85 mmHg)** |  |  |  |  |
| Crude | 1.00 | 0.61 (0.24-1.55) | 0.49 (0.18-1.33) | 0.15 |
| Multivariable-adjusted^2^ | 1.00 | 0.68 (0.24-1.95) | 0.46 (0.14-1.57) | 0.21 |
| **Insulin resistance (HOMA-IR score ≥3.16)** |  |  |  |  |
| Crude | 1.00 | 0.50 (0.20-1.26) | 0.13 (0.05-0.31) | <0.001 |
| Multivariable-adjusted^2^ | 1.00 | 0.73 (0.21-2.56) | 0.12 (0.03-0.48) | 0.01 |

FBS, Fasting Blood Glucose; TG, Triglycerides; HDL, High Density Lipoprotein; BP, Blood Pressure; HOMA-IR, Homeostasis Model Assessment Insulin Resistance.

^1^All values are odds ratios and 95% confidence intervals.

^2^Adjusted for age, gender, energy intake, physical activity levels, socioeconomic status, iron, niacin, saturated fats and BMI.

^3^MDNS components were adjusted for energy intake based on residual method.

^4^HDL-c < 40 mg/dL for the age of < 16 y, and < 50 mg/dL for girls/ < 40 mg/dL for boys in the ages of ≥ 16 y.

**Supplemental Table 3. Multivariable-adjusted odds ratio for MUO (based on IDF criteria) across tertiles of individual methyl donor nutrients (n=203) ^1^**

|  | Tertiles of energy-adjusted nutrient^3^ | | |  |
| --- | --- | --- | --- | --- |
|  | T_1_  (n=67) | T_2_  (n=70) | T_3_  (n=66) | P_trend_ |
| **B2** |  |  |  |  |
| Crude | 1.00 | 0.68 (0.35-1.34) | 0.26 (0.12-0.55) | <0.001 |
| Multivariable-adjusted^2^ | 1.00 | 0.73 (0.30-1.75) | 0.24 (0.08-0.76) | 0.02 |
| **B6** |  |  |  |  |
| Crude | 1.00 | 0.25 (0.12-0.52) | 0.50 (0.25-1.00) | 0.05 |
| Multivariable-adjusted^2^ | 1.00 | 0.39 (0.16-1.00) | 0.91 (0.35-2.32) | 0.86 |
| **B9** |  |  |  |  |
| Crude | 1.00 | 0.37 (0.18-0.74) | 0.30 (0.14-0.61) | 0.01 |
| Multivariable-adjusted^2^ | 1.00 | 0.37 (0.16-0.87) | 0.63 (0.24-1.63) | 0.24 |
| **B12** |  |  |  |  |
| Crude | 1.00 | 0.50 (0.25-1.00) | 0.20 (0.09-0.42) | <0.001 |
| Multivariable-adjusted^2^ | 1.00 | 0.64 (0.27-1.52) | 0.22 (0.08-0.63) | 0.01 |
| **Choline** |  |  |  |  |
| Crude | 1.00 | 0.30 (0.15-0.61) | 0.24 (0.12-0.50) | <0.001 |
| Multivariable-adjusted^2^ | 1.00 | 00.39 (0.16-0.94) | 0.33 (0.13-0.85) | 0.02 |
| **Betaine** |  |  |  |  |
| Crude | 1.00 | 0.76 (0.38-1.52) | 0.92 (0.46-1.82) | 0.81 |
| Multivariable-adjusted^2^ | 1.00 | 0.99 (0.41-2.42) | 1.26 (0.45-3.52) | 0.67 |
| **Methionine** |  |  |  |  |
| Crude | 1.00 | 0.44 (0.22-0.88) | 0.36 (0.18-0.73) | 0.01 |
| Multivariable-adjusted^2^ | 1.00 | 0.58 (0.23-1.44) | 0.36 (0.14-0.96) | 0.04 |

^1^All values are odds ratios and 95% confidence intervals.

^2^Adjusted for age, gender, energy intake, physical activity levels, socioeconomic status, iron, niacin, saturated fats and BMI.

^3^MDNS components were adjusted for energy intake based on residual method.

**Supplemental Table 4. Multivariable-adjusted odds ratio for MUO (based on IDF/HOMA-IR criteria) across tertiles of individual methyl donor nutrients (n=203) ^1^**

|  | Tertiles of energy-adjusted nutrient^3^ | | |  |
| --- | --- | --- | --- | --- |
|  | T_1_  (n=67) | T_2_  (n=70) | T_3_  (n=66) | P_trend_ |
| **B2** |  |  |  |  |
| Crude | 1.00 | 0.63 (0.31-1.26) | 0.32 (0.15-0.68) | 0.01 |
| Multivariable-adjusted^2^ | 1.00 | 0.63 (0.25-1.57) | 0.32 (0.10-1.05) | 0.06 |
| **B6** |  |  |  |  |
| Crude | 1.00 | 0.32 (0.15-0.68) | 0.63 (0.31-1.26) | 0.18 |
| Multivariable-adjusted^2^ | 1.00 | 0.60 (0.23-1.56) | 1.25 (0.47-3.33) | 0.68 |
| **B9** |  |  |  |  |
| Crude | 1.00 | 0.37 (.18-0.76) | 0.32 (0.15-0.66) | 0.01 |
| Multivariable-adjusted^2^ | 1.00 | 0.40 (0.17-0.97) | 0.63 (0.23-1.73) | 0.24 |
| **B12** |  |  |  |  |
| Crude | 1.00 | 0.49 (0.25-0.99) | 0.22 (0.10-0.49) | <0.001 |
| Multivariable-adjusted^2^ | 1.00 | 0.61 (0.25-1.50) | 0.24 (0.08-0.71) | 0.01 |
| **Choline** |  |  |  |  |
| Crude | 1.00 | 0.38 (0.18-0.77) | 0.25 (0.12-0.54) | <0.001 |
| Multivariable-adjusted^2^ | 1.00 | 0.56 (0.23-1.36) | 0.31 (0.11-0.86) | 0.02 |
| **Betaine** |  |  |  |  |
| Crude | 1.00 | 0.85 (0.41-1.77) | 1.20 (0.59-2.42) | 0.63 |
| Multivariable-adjusted^2^ | 1.00 | 1.37 (0.53-3.54) | 2.21 (0.74-6.57) | 0.16 |
| **Methionine** |  |  |  |  |
| Crude | 1.00 | 0.49 (0.24-0.99) | 0.28 (0.13-0.61) | 0.01 |
| Multivariable-adjusted^2^ | 1.00 | 0.80 (0.31-2.05) | 0.26 (0.09-0.74) | 0.01 |

^1^All values are odds ratios and 95% confidence intervals.

^2^Adjusted for age, gender, energy intake, physical activity levels, socioeconomic status, iron, niacin, saturated fats and BMI.

^3^MDNS components were adjusted for energy intake based on residual method.
